# Do New Breeding Techniques in Ornamentals and Fruits Lead to Essentially Derived Varieties?

**DOI:** 10.3389/fpls.2019.01612

**Published:** 2020-03-04

**Authors:** Edgar Krieger, Ellen De Keyser, Jan De Riek

**Affiliations:** ^1^ CIOPORA, The International Community of Breeders of Asexually Reproduced Horticultural Plants, Hamburg, Germany; ^2^ ILVO, Institute for Agricultural and Fisheries Research, Merelbeke, Belgium

**Keywords:** plant breeder’s right, essentially derived varieties, new breeding techniques, UPOV Convention, mutants, fruits, ornamental plants

## Abstract

Do new breeding techniques (NBT) lead to essentially derived varieties (EDV)? It depends! It depends on the definition of EDV in the plant variety right (PVR) laws and their interpretation by the courts. This paper aims at providing an overview of the EDV concept and an analysis of the question whether NBT lead to EDV on the basis of the UPOV 1991 Act, the most recent UPOV Explanatory Notes on EDV of 2017 as well as some selected PVR laws. Almost 30 years ago, the concept of EDV has been incorporated into the UPOV 1991 Act. In order to strengthen the rights of breeders, in particular to provide breeders of original genotypes an additional source of remuneration, a system of “Plant Variety Right specific dependency,” based on “essential derivation,” was developed. Only a very limited number of court cases have been concerned with EDV. However, an escalation in EDV-related conflicts can be expected in the future due to increased competition in the ornamental and fruit breeding business as well as to the application of more sophisticated NBT.

## Introduction

Almost 30 years ago, the concept of essentially derived varieties has been incorporated into the UPOV 1991 Act. In order to strengthen the rights of breeders [UPOV (1989), Introduction, chapter B. 5. (i)], in particular to provide breeders of original genotypes an additional source of remuneration [UPOV (1989), p. 12, No. 6. (iii)], a system of “Plant Variety Right specific dependency (Leßmann, 2000),” based on “essential derivation,” was developed.

Incorporating the EDV concept meant a true extension of the breeder’s rights. The right of the breeder to exclude others from specific acts such as producing, selling, exporting, and importing no longer covers only the protected variety itself, but also varieties that are essentially derived from the protected variety. Thus, the principle of EDV involves questions of the scope of the breeder’s rights and its infringement. It is therefore left to the initiative of the breeders to enforce their rights^1^.

Until now, the EDV concept has been included in the Plant Variety Right laws of 65 UPOV member states, 7 of them being party only to the UPOV 1978 Act^2^. Unfortunately, the Plant Patent Act of the United States, the basic and most widely used regulation for the protection of intellectual property of ornamental and fruit breeders in the U.S., does not yet contain a provision on EDV^3^.

Several publications about EDV have been issued^4^. Only a very limited number of court cases have been concerned with EDV^5^, apparently due to the fact that breeders are hesitant to start court proceedings due to the complexity of the matter and the ambiguous provisions of the applicable plant variety right (PVR) laws. Additionally, breeders seem to be more careful in their breeding programs in order to avoid possible EDV cases. At least in the US (ASTA) and France (SEPROMA), for maize, different zones (red, orange, green) have been agreed upon when dealing with pairwise genetic distances between potential initial and essentially derived varieties based on molecular marker profiles. It has become part of the arbitration system but is also used in practice to keep away from breeding too close to a competitor’s genetic material (UPOV-BMT reports, available on https://www.upov.int/portal/index.html.en).

**Figure 1:**
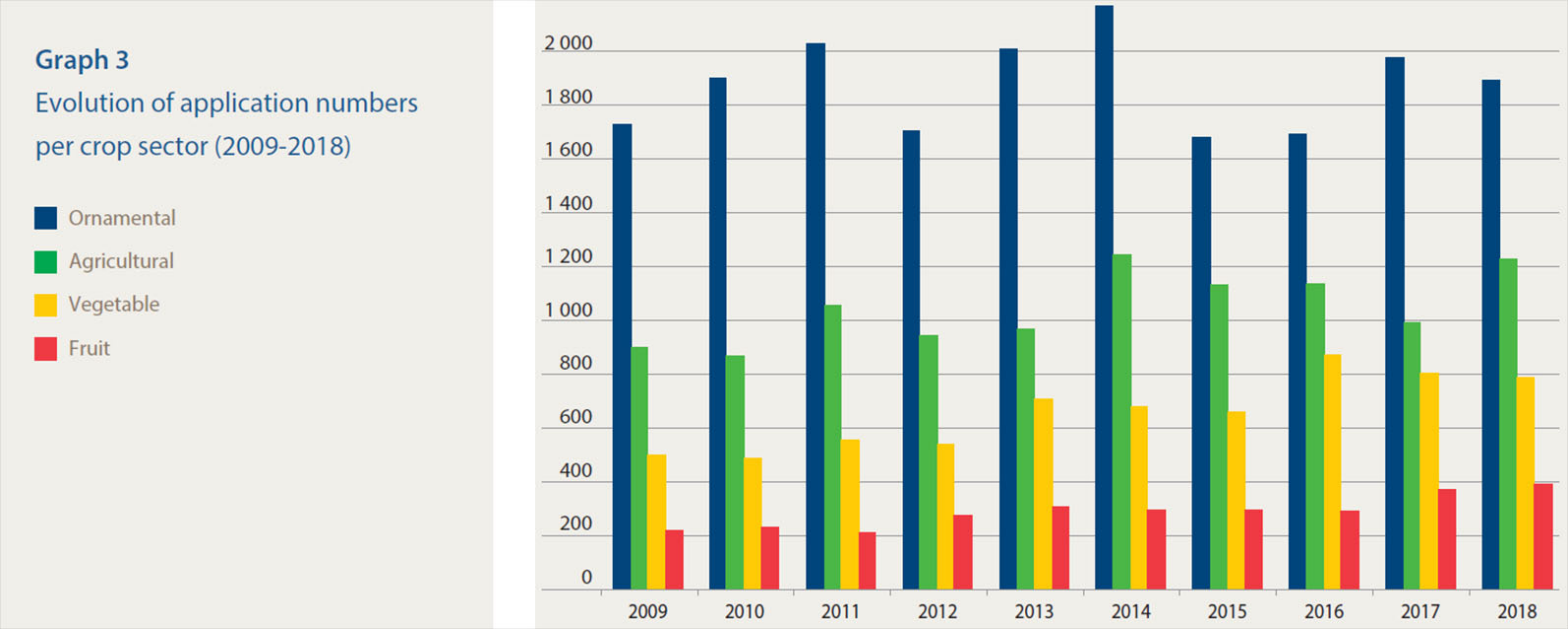
Evolution of PBR-application numbers per crop sector (2009–2018) at the CPVO (2018).

However, an escalation in EDV-related conflicts can be expected in the future due to increased competition in the ornamental and fruit breeding business as well as to the application of more sophisticated “breeding” methods, the so-called new breeding techniques (NBT). In ornamentals and fruits, one is typically dealing with long breeding times that can reach up to 20 years. However, mutations into these crops are easily detected when propagated on larger scales and can immediately be introduced into the market. By use of NBT, targeted development of innovative EDV can become a very attractive and fast route to new varieties. Different to seed propagated species where the owner of a variety often also controls the propagation and final marketing of the seeds, vegetative species are in a way “free” and have to rely on a stronger IP protection system. The PBR system in Europe is seen by the sector as one of the most performant systems, and this results into a high number of applications in ornamental and fruit crops (**Figure 1**) (CPVO, 2018).

**Figure 2:**
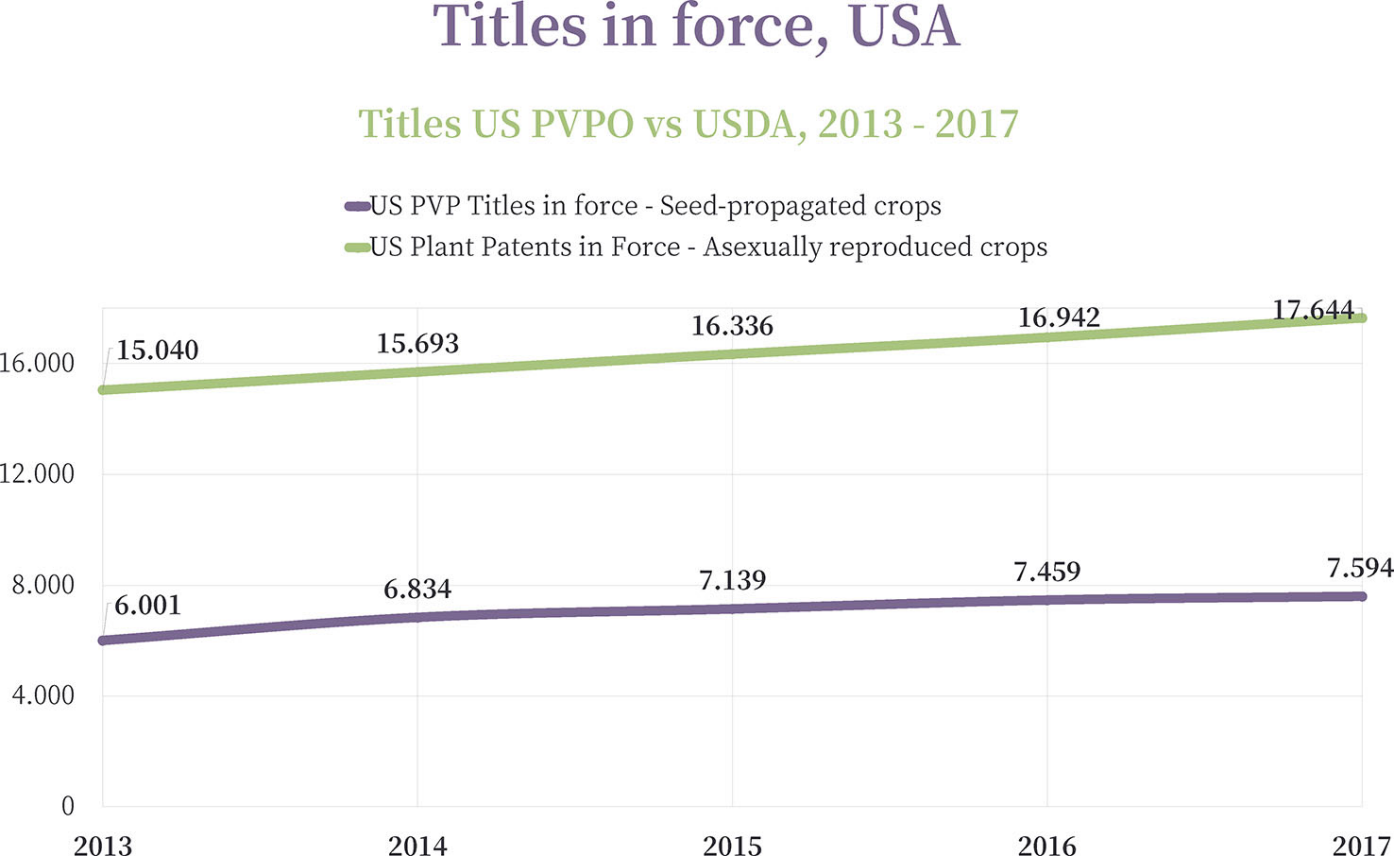
Evolution of the U.S. Plant Variety Rights (PVP) vs. U.S. Plant Patents in force in each consecutive year (2013–2017). Source: UPOV, 2019.

**Figure 3:**
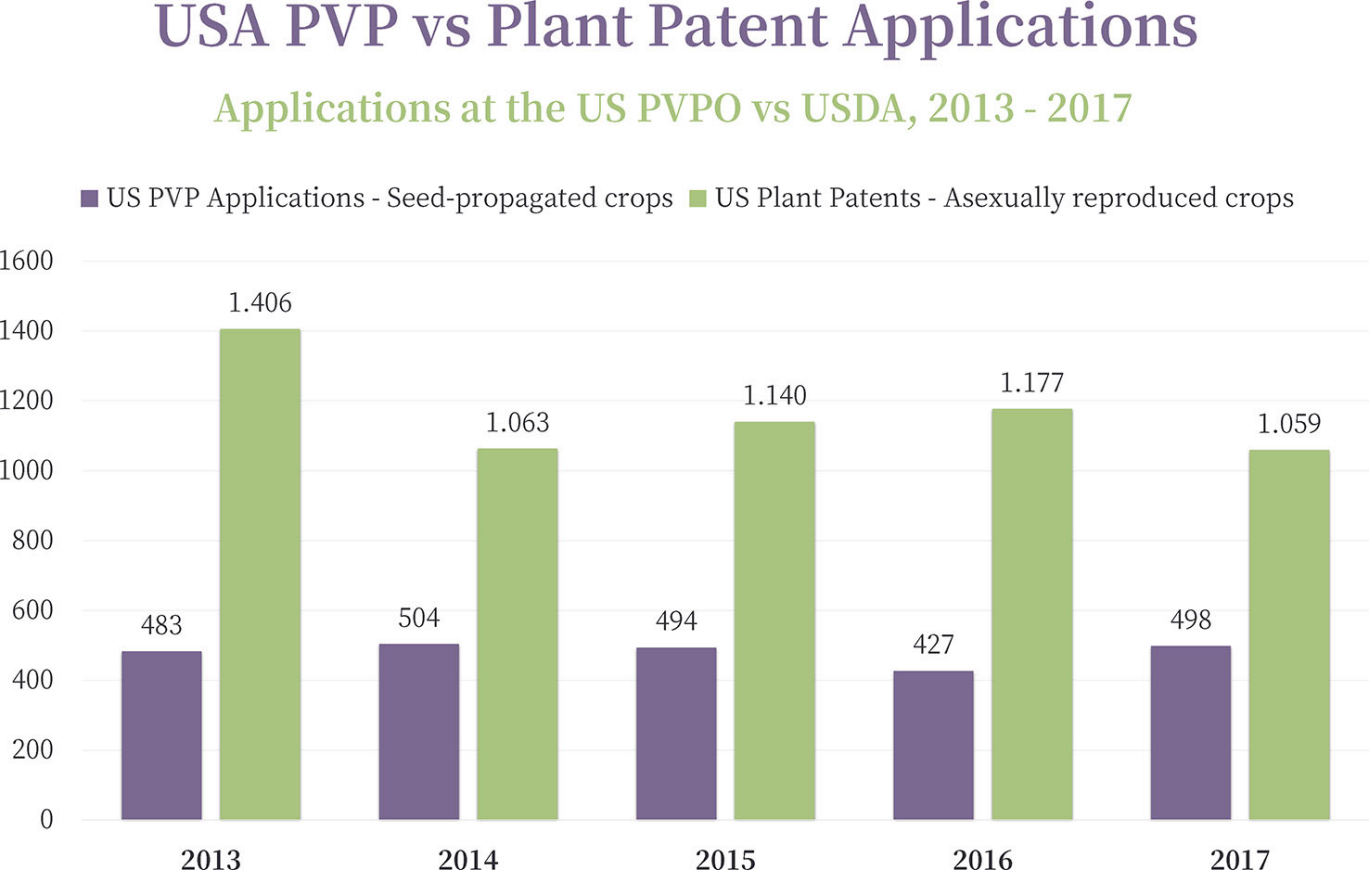
Evolution of the U.S. Plant Variety Rights (PVP) vs. U.S. Plant Patent applications (2013–2017). Source: UPOV, 2019.

In the United States, two main IP systems are used for the protection of plant Fi varieties: Plant Patents for the asexually reproduced plants (other than tuber-propagated) and Plant Breeders´ Rights for the seed propagated crops^6^ (**Figure 2**). Over the past 5 years, twice as many applications were filed for Plant Patents than Plant Breeders’ Rights in the U.S. (**Figure 3**) demonstrating the significance of IP protection for asexually reproduced species.

According to the High Level Group of Scientific Advisors (2017), the term NBT describes a very diverse range of techniques, some of which are substantially different from established transgenic approaches in their way of introducing traits to an organism (EASAC, 2015). Some are a refinement of conventional breeding techniques and insert genetic material that is derived from a sexually compatible species, while some nevertheless are used in combination with established techniques of genetic modification. Some of the NBT result in organisms that contain only point mutations and are practically indistinguishable from varieties bred through conventional breeding methods or resulting from spontaneous mutations (EASAC, 2015). In this paper we focus on NBTs (as listed by the EU and in detail explained by the High Level Group of Scientific Advisors, 2017), but explicitly not including “grafting” and “agro-infiltration,” as the resulting products of these above defined NBTs are most similar to conventional mutagenesis and genetic modification.

## The Text of the UPOV 1991 Act Regarding EDV

The starting point of this discussion about EDV is the text of the UPOV 1991 Act. However, it must first be noted that the UPOV 1991 Act, like any other UPOV Act, does not have a direct effect in the UPOV member states. The UPOV Act only sets the minimum requirements of PVR laws in the UPOV member states. The legal basis for EDV are the specific PVR laws on which the PVR title and the EDV-claim are based. In many cases, the wording of the provisions dealing with EDV of such PVR laws differs significantly from the wording of the UPOV 1991 Act ^7^. However, it is solely the provisions of the applicable PVR laws that govern the legal relationship of those involved. Only when there are incomplete or inconclusive provisions that give room for interpretation, the UPOV text may be consulted. In this regard, UPOV has since 2008 drafted two explanatory notes (EXN) on EDV, which aim at providing guidance and assist members of UPOV and relevant stakeholders in their consideration in matters concerning EDV (UPOV, 1992; UPOV, 2017). The most recent one is the EXN on EDV approved in April 2017. However, it should be noted that the EXN is not binding for UPOV members and must not be interpreted in a way that is inconsistent with the relevant UPOV Act.

The sections regarding EDV can be found mainly in Article 14 (5) (a) (i), (b), and (c) of the UPOV 1991 Act. They have to be read in the context of the complete Article 14 (Scope of the Breeders´ Rights), in which the provisions have been incorporated. Additionally, Article 15 (1) (iii) also contains an important link to EDV.

**Article 14**

**Scope of the Breeder’s Right**

(1) [*Acts in respect of the propagating material*]

(2) [*Acts in respect of the harvested material*]

(3) [*Acts in respect of certain products*]

(4) [*Possible additional acts*]

(5) [*Essentially derived and certain other varieties*]

*(a)* The provisions of paragraphs (1) to (4) shall also apply in relation to

(i) varieties which are essentially derived from the protected variety, where the protected variety is not itself an essentially derived variety,

(ii) varieties which are not clearly distinguishable in accordance with Article 7 from the protected variety and

(iii) varieties whose production requires the repeated use of the protected variety.

*(b)* For the purposes of subparagraph *(a)* (i), a variety shall be deemed to be essentially derived from another variety (“the initial variety”) when

(i) it is predominantly derived from the initial variety, or from a variety that is itself predominantly derived from the initial variety, while retaining the expression of the essential characteristics that result from the genotype or combination of genotypes of the initial variety,

(ii) it is clearly distinguishable from the initial variety and

(iii) except for the differences which result from the act of derivation, it conforms to the initial variety in the expression of the essential characteristics that result from the genotype or combination of genotypes of the initial variety.

*(c)* Essentially derived varieties may be obtained for example by the selection of a natural or induced mutant, or of a somaclonal variant, the selection of a variant individual from plants of the initial variety, backcrossing, or transformation by genetic engineering.

**Article 15**

**Exceptions to the Breeder’s Right**

(1) [*Compulsory exceptions*] The breeder’s right shall not extend to

(i) acts done privately and for non-commercial purposes,

(ii) acts done for experimental purposes and

(iii) acts done for the purpose of breeding other varieties, and, except where the provisions of Article 14 (5) apply, acts referred to in Article 14 (1) to (4) in respect of such other varieties.

## The Basics of “Breeder’s Exemption” and “Dependency” and the Historical Background and Motives for Incorporating the EDV-Concept Into the UPOV 1991 Act

### Free Access to Germplasm

From the very first day of the UPOV-system, starting with the UPOV 1961 Act, the principle of free access of breeders to existing breeding material (genotypes/germplasm) for the purpose of breeding new varieties has been established. The main reason for having included and maintained this principle through the UPOV Acts is that any breeding is based on existing living material and thus breeders have to depend on free access to different genotypes to avoid a concentration on only a very limited number of varieties (Leßmann, 2000). The majority of breeders and their associations support this principle of free access to germplasm (see CIOPORA).

This is a fundamental element of the UPOV system of plant variety protection known as the “breeder’s exemption,” whereby there are no restrictions on the use of protected varieties for the purpose of breeding new plant varieties. The authorization of the breeder for the use of protected varieties for breeding purposes is required neither under the 1978 Act nor under the 1991 Act. The Breeder’s Exemption also permits the application of NBT on protected varieties.

There is no such concept of breeder’s exemption in the patent system under the European Patent Convention (EPC) although it has been implemented in some national patent laws. Still breeders also take into account patent protection because of the innovations involving technical solutions to develop new varieties. Under the current European patent protection, which is a stronger and more absolute protection than PVR in respect of the strict substantial requirements examination and its scope of exclusive rights, any use of the patented products (e.g. genetic material) and processes covered by the patent must obtain permission from the patent owner. This blocks access to biological materials for further breeding.

### The Shortcomings of the Overly Broad “Breeder’s Exemption” in the Past

Compared to the position of a patent holder as described before, the position of breeders with regard to the scope of protection was weak before the revision of the UPOV Act in 1991. Additionally, too many loopholes where found in the individual PVR laws. In particular the broad wording of the “breeder’s exemption” combined with the absence of adequate provisions on the control of varieties that are very similar compared to a protected variety left the door wide open to so called “cosmetic breeding” and plagiarism. In addition, the situation regarding “mutants” was not satisfactorily covered by the PVR laws, as the breeders of the original varieties were unable to control said mutants. Even by way of license-agreements, an adequate control could not be reached (decision of the European Commission, 1985).

### Mutants and New Bio-Technologies as the Initial Point for the EDV-Concept

Therefore, it was mainly the breeders of vegetatively reproduced crops, namely ornamental and fruit plants, who were dissatisfied with the fact that third parties, due to the limited scope of protection of the preceding UPOV Acts, were allowed to exploit and even acquire PVR protection for mutants of protected varieties without the original breeder being able to participate in the use and exploitation of these mutants (Kiewiet, 2002). Mutants play a significant role in many ornamental species. As estimated by breeding companies, many of important ornamental varieties are mutants:

In addition, it was the development of GMO technologies which enables adding new characteristics to existing varieties by way of biotechnological methods that led to the introduction of the concept of dependent plant variety rights. Conventional breeders were concerned that such new GMO varieties could be used without them receiving any financial compensation for the use of the germplasm they have created through conventional breeding methods.

This discussion has gained momentum again in the recent past with the advent of NBT. Conventional GMO have not been applied in ornamentals and fruits a lot due to high costs of regulatory issues and companies not willing to put their reputation at risk. Mutagenesis on the contrary has yielded more than 3,200 novelties, some of them well known like the orange flesh grapefruit (Source FAO/IAEA Mutant Varieties Database: https://mvd.iaea.org/). NBT also allow to develop a trait in a parental line that can be quickly introgressed by backcrossing into an existing variety. In species with a short commercial life, like lettuce and other vegetables, this new phenomenon might lead to even more closely related varieties. Again, the fear is raised that an NBT variety could easily take over the market when an innovative feature is added onto a conventionally bred variety.

## Analysis of the EDV-Concept

### Systematic Framework

The EDV clause is included in Article 14 of the UPOV 1991 Act (“Scope of the Breeder’s Right”). This shows that the EDV concept is part of the scope of the right and not an exception or limitation, like those provisions of Article 15 of the 1991 Act.

However, the EDV Concept is a limitation in another sense: According to Article 15 (1) (iii) *the breeder’s right shall not extend to acts done for the purpose of breeding other varieties, and, except where the provisions of Article 14 (5) apply, acts referred to in Article 14 (1) to (4) in respect of such other varieties (Breeder’s´ exemption)*. In other words: Acts for the purpose of breeding a new variety are always allowed without the consent of the breeder of the initial variety, but the exploitation of such a new variety is allowed only as long as the new variety is not considered to be an EDV. In that way the EDV Concept is indeed a limitation of the breeders´ exemption, not in respect of the free access to germplasm, but in respect of the commercialization of the newly developed variety, if this is an EDV.

### “Classical Breeding Work” in the EDV Concept

The term “classical breeding work”^8^ is one of the keywords in the EDV concept. The term first leads to a discussion on the contradiction between the definition of “breeder” in Article 1 (iv) of the UPOV 1991 Act and the “classical breeding work” in the framework of the EDV concept. Whereas according to Article 1 (iv) of the UPOV 1991 Act both, i.e., those who “cross and select” as well as those who “discover and develop,” new varieties deserve the title “breeder,” in the EDV-concept “classical breeding” only means the crossing of parental varieties and the selection of the resulting progenies with the aim to create new variations. The definition of a “breeder” mainly results from the purpose of the UPOV 1991 Act to be applied also to a variety originating from a mutation (see UPOV, 1991). However, although UPOV does not differentiate between “classical breeding work” a “discovering and developing” on the level of the definition of the term “breeder,” UPOV nevertheless sets apart the “classical breeding work”, as only the results of such “classical breeding work” shall benefit from the EDV concept and from the extension of the scope of rights. The reason for this is that huge personal and financial endeavors have to be made to create new varieties by way of such “classical breeding”.

In many parts of the world, breeders are often small and medium-sized companies. Until now they mostly breed innovative varieties in a conventional way by crossing and selection, which can take up to 20 years of hard work. Breeding innovative varieties in a conventional way is one of the backbones of the ornamental and fruit industries. It requires significant human and financial investment to develop such varieties. In order to guarantee a sustainable continuation of such breeding there needs to be a sufficient return on investment. Only varieties that are the result of classical breeding work qualify for the extended protection provided by the EDV concept.

### The Conditions of an EDV

#### The Initial Variety (Article 14 (5) (a) (i) UPOV 1991 Act)

The initial variety forms the basis of any EDV claim. It derives from the principle of dependency that the initial variety must enjoy PVR protection or at least provisional protection according to Article 13 of the UPOV 1991 Act. Therefore, in general the dependency of an essentially derived variety starts with the beginning of the provisional protection of the initial variety and ends with the end of protection of the initial variety (either by expiration or cancellation). Additionally, the initial variety cannot itself be an EDV. Although by introducing the EDV concept a certain degree of dependency has been created, so called “dependency pyramids” were to be avoided. The initial variety, therefore, must be the result of “classical breeding work” [UPOV, Doc. IOM/IV/2, page 12, No. 6 (iv)]. As already mentioned before, essential derivation is a matter of fact. Therefore, an EDV remains an EDV forever. Even if the protection period of the initial variety is exhausted, all varieties derived from this initial variety will still be essentially derived from the initial variety, but not *dependent* of the initial variety which is no longer protected. The reason for this is that the EDV-concept has mainly been introduced to protect more efficiently the breeder of the initial variety and not those who make derivations from his work (see also International Seed Federation, 2005).

#### Clearly Distinguishable

The EDV has to be *clearly distinguishable* from the initial variety. This requirement draws the line between an EDV and a variety which is not clearly distinguishable from the protected variety in the meaning of Article 14 (5) (a) (ii) in combination with Article 7 UPOV 1991 Act (see UPOV, 2017). Whereas the EDV is a discrete variety which is in principle eligible for PVR protection^9^, a variety not clearly distinguishable from the protected variety is not a discrete one and cannot enjoy separate PBR protection but falls automatically within the scope of the earlier protected variety.

Some claim that the EDV concept aims at preventing plagiarism. However, in our view plagiarism is not a question of derivation or dependency, but rather a question of Minimum Distance/Distinctness and direct infringement. If a variety in its phenotype very much resembles a protected variety, it is not clearly distinguishable from the protected variety, and its commercialization is a direct infringement, irrespective whether the new variety is (essentially) derived from the protected variety or not. Instead, the fact that an EDV needs to be distinct from its Initial Variety makes it clear that a plagiaristic variety can never be regarded as EDV, as a plagiaristic variety already lacks the Distinctness. Declaring plagiaristic varieties as EDV would have the strange consequence that PBR Offices would be forced to grant Plant Breeders´ Rights titles to plagiaristic varieties, because EDV in principle are eligible for PVR protection.

The application of NBT usually will result in varieties which are clearly distinguishable from their Initial Variety. In fact, such varieties usually would not aim at copying an existing variety but adding an important or innovative trait to the initial variety. NBT are in principle not plagiaristic.

#### Predominant Derivation

The second condition an EDV will have to fulfil as stipulated by the UPOV 1991 Act is that it is *predominantly derived* from the initial variety or, as the case may be, from a variety that itself is *predominantly derived* from the initial variety. Predominant derivation relates to the genetic origin of the variety.

The first and in the field of vegetatively reproduced ornamental and fruit varieties by far the most important group of EDV are so called mono-parental varieties, like mutations, that are not only predominantly, but totally derived from their mother-variety. The importance of this group of varieties is mirrored by the examples given as acts of “derivation” in Article 14 (5) (c) UPOV 1991 Act. Four of the examples listed in the UPOV Act, i.e., mutants, somaclonal variants, variant individuals from plants of the initial variety, and genetically modified plants (GMO) resulting from transformation by genetic engineering, are mono-parental varieties. A mono-parental variety has its basis in one genome only (the genome of the initial variety), which was altered by the acts of derivation mentioned before. The half-sentence “while retaining the expression of the essential characteristics that result from the genotype or combination of genotypes of the initial variety” indicates that predominantly derived varieties essentially retain the expressed characteristics of the initial protected variety but does not stipulate an additional requirement for predominant derivation. The meaning of this sentence is further limited by (iii): “except for the differences which result from the act of derivation.”

For NBT varieties the condition of predominant derivation is fulfilled, because NBT varieties—like mutants—are mono-parental varieties, solely derived from their Initial Variety. Even if by way of the NBT multiple changes are made to the genome of the initial variety (stacking), the new variety is based solely on the genome of the initial variety and the genetic conformity will be very high.

#### Conformity to the Initial Variety

The main dispute in regard to EDV is about the alleged requirement of conformity of the EDV compared to its Initial Variety. A judgment on the question on the degree of conformity must be reached on the basis of the expression of characteristics which result from the genotype of the initial variety. This judgment has to assess the conformity to the description of the initial variety apart from the specific differences which result from such breeding methods and other minimal differences which result incidentally from such breeding methods, such differences being evidenced at the level of the genome, the genotype or the phenotype. Article 14(5)(b)(iii) does not set a limit to the amount of difference which may exist where a variety is considered to be essentially derived. Differences, which result from the act of derivation, shall not be taken into consideration for the determination of an EDV.

Voices in literature are of the opinion that only varieties that show one or, at the most, a very limited number of phenotypic differences, can be considered as EDV (without reasoning, van der Kooij (1997), Introduction to the EC Regulation on Plant Variety Protection, Art. 13 (5) EC Regulation 2100/94, page 32; Court of Civil Law in The Hague, footnote 8, which based this opinion on its interpretation of the term “essentially derived” in indent (i) of Article 14 (5) (b) UPOV 1991 Act and UPOV document IOM/IV/2, page 12, No. 6 (ii), without considering the later UPOV Doc IOM/6/2. In an even more narrow interpretation, the UPOV EXN on EDV of 2017 states that a variety cannot be predominantly derived if it does not retain the essential characteristics of the initial variety [see UPOV (2017)]. This sentence is interpreted in a way that a variety can be considered to be an EDV only if it retains all essential characteristics of its Initial Variety. The EXN follows the very narrow Australian approach on EDV, where the Australian PBR Office declares a variety as EDV only if it differs in an unessential characteristic from the Initial Variety (see presentation of Australia in UPOV EDV Seminar 2013, https://www.upov.int/edocs/mdocs/upov/en/upov_sem_ge_13/upov_sem_ge_13_ppt_9.pdf).

We do not agree to this approach. Already the wording of Article 14 (5) (a) (ii) shows that this argument is not cogent. The condition “clearly distinguishable” according to Article 7 of the UPOV 1991 Act requires at least one “clear” difference between the EDV and the initial variety, whereas in several cases even one difference is not enough to consider one variety “clearly” distinguishable from another [see e.g. Article 5.3.3.2.1 of the UPOV document TG 1/3 “General Introduction to the Examination of Distinctness, Uniformity and Stability and the Development of Harmonized Descriptions of new Varieties of Plants” https://www.upov.int/tgp/en/introduction_dus.html)].

The narrow interpretation of the EDV-concept in allowing only one or fewer differences between the initial variety and its EDV disregards the new tendencies in the development of new varieties, because certain methods of developing new varieties, applying chemicals and other mutagens or NBT, allow the development of plants which differ considerably from the mother plant without altering the genome of the plant significantly. In fact, depending on the act of derivation the number of differing phenotypic characteristics between the initial variety and the variety derived thereof can differ significantly, between one, a few or even numerous. For example, mitotic polyploids express in general an increased size in all plant organs and an intensification of physiological characters. Such increases in plant organs can easily lead to numerous different characteristics as described in the test guidelines provided for by UPOV.

**Figure 4:**
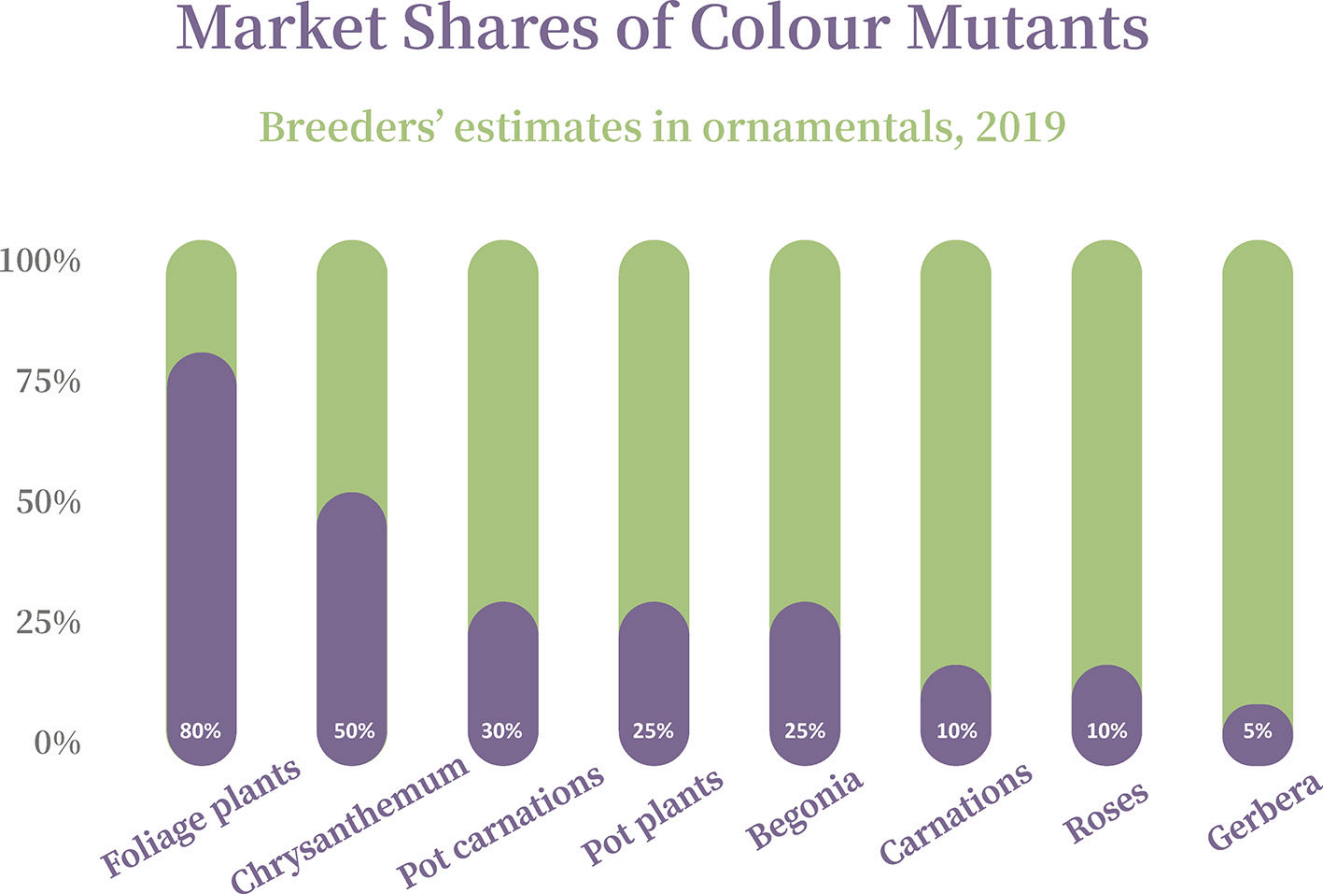
Market shares of color mutants in ornamentals as estimated by breeders, 2019.

Additionally, requiring that an EDV must retain all essential characteristics of the Initial Variety would make the EDV Concept meaningless to a huge extent. A flower color-mutant in an ornamental variety is one typical case of an EDV. The characteristic “colour” can be regarded as one of the most if not the most important characteristics in ornamental varieties (**Figure 4**), presumably an *essential* characteristic. The colour-mutant clearly does not retain the essential characteristic “colour” of the initial variety and thus could not be considered an EDV, although being a mutant, is *the* typical example of an EDV and has been one of the main reasons for the introduction of the EDV-concept^10^.

Also varieties resulting from NBT do in principle not retain all essential characteristics of their Initial Varieties, because the NBT have been deliberately applied with the aim to change essential characteristics of the initial variety, e.g. by introducing a resistance into a susceptible variety or to limit the browning of apples (“Arctic Apple”, https://www.arcticapples.com/). According to the narrow UPOV EXN on EDV of 2017 and the Australian approach (Government of Australia, 2002), such NBT varieties would not be considered EDV. However, against the background that the NBT variety consists almost entirely of the genome of the initial variety, it seems highly unfair to the breeder of the initial variety to deprive him of any benefit from the NBT. Additionally, if the New Breeding Technology is protected by a Patent, the Patent holder can prevent the breeder of the initial variety from commercializing his variety or even to further breed with it. In order to prevent such a situation (in 1991 with a focus on GMO), the EDV Concept was established.

The narrow approach of the UPOV EXN on EDV is based on the last half sentence of indent (i) of Article 14 (5) (b) of the UPOV 1991 Act, which reads:

A variety shall be deemed to be essentially derived from another variety (“the initial variety”) when

- it is predominantly derived from the initial variety, or from a variety that is itself predominantly derived from the initial variety, while retaining the expression of the essential characteristics that result from the genotype or combination of genotypes of the initial variety,.

This further requirement in indent (i) is inconsistent with indent (iii) (Kiewiet, 2002). To avoid this inconsistency, the Community Plant Variety Right Regulation 2100/94 of 27 July 1994^11^, which is based on the UPOV 1991 Act, contains a definition of EDV that has not taken over the last part of Article 14 (5) (b) (i) UPOV 1991 Act.

6. For the purposes of paragraph 5 (a), a variety shall be deemed to be essentially derived from another variety, referred to hereinafter as ‘the initial variety’ when:

- it is predominantly derived from the initial variety, or from a variety that is itself predominantly derived from the initial variety;
- it is distinct in accordance with the provisions of Article 7 from the initial variety; and
- except for the differences which result from the act of derivation, it conforms essentially to the initial variety in the expression of the characteristics that results from the genotype or combination of genotypes of the initial variety.

By doing so, the Community PVR system, one of the largest systems under the regime of UPOV, also has obviously put the focus on the genetic conformity between an EDV and its initial variety. This definition of EDV has also been incorporated in the PVR laws of Bulgaria, Czech Republic, Estonia, France, Germany, Romania and Slovenia^12^.

Therefore, the focus of the discussion should be on indent (iii) of Article 14 (5) (b) of the UPOV 1991 Act. Indent (iii) stipulates that the EDV shall *conform to the initial variety in the expression of the essential characteristics that result from the genotype or combination of genotypes of the initial variety, except for the differences which result from the act of derivation*. Indent (iii) does not set a limit to the number of phenotypic differences between an EDV and the initial variety to one or a few [this is explicitly pointed out in the UPOV (1989) Doc. IOM/6/2, page 4, No. 12]. According to indent (iii) a variety shall be considered an EDV as long as its differences with the initial variety result from the act of derivation.

As far as vegetatively reproduced ornamental and fruit varieties are concerned it can be taken for granted that all phenotypic differences between a mutant and its mother variety result from the act of derivation.

When dealing with the regulatory aspects of NBTs, the High Level Group of Scientific Advisors (2017) dealt with spontaneous mutation, induced mutagenesis and genome editing technologies. In summary, they conclude: “The spontaneous mutation rate is about 7 x 10^-9^ base substitutions, per site, per generation. This results in one base substitution per generation in a genome the size of *Arabidopsis thaliana* (Ossowski et al., 2010). This means that unintended effects can also accumulate in sexual crossing. Induced mutagenesis, depending on intensity and concentration of the mutagenic agent, can increase this mutation rate by a factor of approximately 500 (Jander et al., 2003; Till et al., 2007; Cooper et al., 2008). All mutations occurring in addition to the mutations conferring the desired trait can be considered ‘off-target’. There is a high probability that the random mutations in some genes will also influence the expression of other genes. Typically, however, the selected plants with the desired traits will still contain a high number of undetected random mutations, in particular if they do not cause disadvantageous phenotypic traits (Acquaah, 2015; Popova et al., 2015). Consequently, the breeder must undertake time consuming downstream selection in order to identify the desired traits essentially on the basis of the phenotype. This selection process does not exclude the presence of unidentified mutations in the new variety. The use of the new techniques involving ODM (oligo-directed mutagenesis) and SDN (site-directed nucleases) implies a different strategy. In this case the number of mutations is greatly reduced by comparison with the above and is limited to one or a few predefined mutations and possibly some off-target mutations.”

Nevertheless, in general a mutant will also and always retain several important characteristics of its mother variety, because it was the very reason for the developer of the mutant to benefit from these important characteristics of the mother variety – this was the very reason why he had chosen this mother variety, and not e.g. a free, older variety.

## Conclusion

After all, first generation varieties resulting from New Breeding Techniques, are mutants and thus are solely derived from their Initial Variety. This can have a direct impact on vegetatively propagated plants like ornamentals and fruits as existing varieties immediately can be improved by NBT. Therefore, for these crops, direct NBT varieties should always be considered EDV. However, applying NBT in other cultivated crops breeding activities can result in more genetically narrow varieties too. First, the EDV concept is valid for all crops already although the delineation in seed crops is less straightforward than in vegetatively propagated plants. The high genetic conformity, which is obvious for mutants in fruits and ornamentals, needs then to be defined, mostly by using conformity measures based on an agreed set of molecular markers (in future this might become DNA sequence homology). Agreements on thresholds for genetic conformity have been made between breeders of certain species already. At the moment it merely deals with recurrent backcrossing and not too much with mutation breeding or use of NBTs. However, it can be expected that e.g. targeted mutagenesis by CRISPR/Cas and a short recurrent backcrossing cycle might become a future strategy in rapid cycling varieties e.g. vegetable breeding. If there continues to be an imbalance between the breeder of the initial variety that has created a new beneficial “mix” of genetic diversity within a genotype and NBT breeders that add selected improvements on existing varieties, an erosion and narrowing of genetic diversity could be the result.

A too narrow interpretation of the EDV Concept, as currently applied by the Australian Government, deprives breeders of initial variety from effective protection and an additional income. It fails to meet the aim of the EDV Concept as implemented in the UPOV 1991 Act, but rather steps back to the scope of the UPOV 1978 Act.

## Author Contributions

EK described the legal aspects of EDV within the UPOV Acts and member state legislations. EDK contributed the technical explanations on NBT, and JDR coordinated the writing of the paper and reviewed it.

## Conflict of Interest

The authors declare that the research was conducted in the absence of any commercial or financial relationships that could be construed as a potential conflict of interest.

## Footnotes

1 A different approach is—yet—followed by Australia. Australia is—as far as can be seen—the only UPOV member state where the PVR authorities play an active role in the declaration of a new variety to be or not to be an EDV.

2 Bolivia, Brazil, Colombia, Ecuador, Italy, Nicaragua, and South Africa.

3 Since 2018 the US Plant Variety Protection Act is open for vegetatively reproduced crops, too, so that the breeders of such crops can benefit from the EDV Concept in the USA, too.

4 See list of literature at the end of the article for Crespel et al. (2009), Hunter (1999), Korzun and Heckenberger (2004), Lange (1993), Vosman et al. (2004) and Zhang et al. (2001).

5 The Court of Civil Law in The Hague, The Netherlands (File-No. 2003/1054) on 13 July 2005 has issued a decision in a case concerned with Gypsophila and denied the existence of an EDV, the Tel Aviv-Jaffa District Court, Israel (File - No.002002/05) on 21 September 2005 approved for the same variety the existence of an EDV. See also Turin Trial Court, File-No GR No 28969/2009, Judgment No 3519/2015 published on 14/05/2015, Regional Court of Mannheim, decision 7 O 442/04.

6 Since 2019 asexually reproduced crops can also be protected by Plant Breeders´ Rights—driven by the missing EDV concept in the Plant Patent Act. Additionally, varieties can be protected by Utility Patents in the US, too, but this was not common practice for asexually reproduced crops in the past.

7 The majority of the 65 member states of UPOV with an EDV provision have strictly copied the UPOV text on EDV. A prominent example for a different wording is the European PVR-Regulation 2100/94.

8 Or “true breeding work” as used in UPOV (1989).

9 If it meets the additional criteria (novelty, uniformity and stability).

10 In fact, there are voices which deny the existence of an EDV in such case, see the citation of Kiewiet (2002) in: Plant Variety Rights in a community context, page 5, Opposite to this, the case of a colour mutation is listed as one example of an EDV in the brochure of the Japanese PVR office explaining the PVR system in Japan.

11 Published in all EC-languages under www.cpvo.eu.int.

12 The English texts are published under www.upov.int.

## References

[B1] Acquaah G. (2015). “Conventional Plant Breeding Principles and Techniques,” in Advances in Plant Breeding Strategies: Breeding, Biotechnology and Molecular Tools, 115–158. 10.1007/978-3-319-22521-0_5

[B2] CIOPORA Position on Breeders’ Exemption of April 2014 https://www.ciopora.org/ciopora-position-papers.

[B3] Cooper J. L. Till B. J. Laport R. G. Darlow M. C. Kleffner J. M. Jamai A. (2008). Tilling to detect induced mutations in soybean. BMC Plant Biol. 8 (1), 9. 10.1186/1471-2229-8-9 18218134 PMC2266751

[B4] CPVO (2018). Annual report. https://cpvo.europa.eu.

[B5] Crespel L. Pernet A. Le Bris M. Gudin S. Hibrand Saint Oyant L. (2009). Application of ISSRs for cultivar identification and assessment of genetic relationships in rose. Plant Breed. 128 (5), 501–506.

[B6] EASAC (2015). New breeding techniques. European Academies’ Science Advisory Council For. Retrieved from http://www.easac.eu.eu.

[B7] European Commission (1985). 85/561/EEC: Commission Decision of 13 December 1985 relating to a proceeding under Article 85 of the EEC Treaty (IV/30.017 - Breeders' rights: roses). http://data.europa.eu/eli/dec/1985/561/oj.

[B8] Government of Australia (2002). Clarification of plant breeding issues under the Plant Breeder’s Rights Act 1994. Rep. Expert Panel Breed., 19–41. www.anbg.gov.au/breeders/essentially-derived-variety.html.

[B9] High Level Group of Scientific Advisors (2017). New Techniques in Agricultural Biotechnology, Explanatory Note 02, https://ec.europa.eu/research/sam/pdf/topics/explanatory_note_new_techniques_agricultural_biotechnology.pdf, ISBN 978-92-79-66222-5, 10.2777/574498, KI-02-17-242-EN-N.

[B10] Hunter R. B. (1999). Essentially Derived and Dependency, some examples, Intellectual Property Committee, CSTA, Vancouver BC, July 20, 1999.

[B11] International Seed Federation (2005). Essential Derivation - Information and Guidance to Breeders 1–10.

[B12] International Seed Federation (2012). View on IP.

[B13] Jander G. Baerson S. R. Hudak J. A. Gonzalez K. A. Gruys K. J. Last R. L. (2003). Ethylmethanesulfonate saturation mutagenesis in Arabidopsis to determine frequency of herbicide resistance. Plant Physiol. 131 (1), 139–146. 10.1104/pp.102.010397 12529522 PMC166794

[B14] Kiewiet B. (2002). Plant Variety Rights in a Community context. Speech made at the occasion of a symposium organised by the “Vereniging voor Agrarisch Recht” on 11.9.2002, 1–6. https://www.genres.de/fileadmin/SITE_MASTER/content/Schriftenreihe/Band24_Gesamt.pdf.

[B15] Korzun V. Heckenberger M. (2004). Methodik zur Bestimmung im wesentlich abgeleiteter Sorten (Identification of essentially derived varieties): 7–12. In Analyse und Bewertung der genetischen Vielfalt in der Land-, Forst- und Fischereiwirtschaft zur Ableitung von Entscheidungskriterien für Erhaltungsmaßnahmen Tagungsband eines Symposiums am 27 September 2004 in Mareinsee, Neustadt a. Rbge.

[B16] Lange P. (1993). Abgeleitete Pflanzensorten und Abhängigkeit nach dem revidierten UPOV-Übereinkommen. GRUR Int. 1993 (2), 137–143.

[B17] Leßmann H. (2000). Weiterzüchtung und Sortenschutz - Entwicklung der gesetzlichen Regelung, Festschrift for Rudolf Lukes.

[B18] Ossowski S. Schneeberger K. Lucas-Lledó J. I. Warthmann N. Clark R. M. Shaw R. G. (2010). The rate and molecular spectrum of spontaneous mutations in Arabidopsis thaliana. Sci. (New York N.Y.) 327 (5961), 92–94. 10.1126/science.1180677 PMC3878865 20044577

[B19] Popova E. Mukund S. Kim H. H. Saxena P. K. (2015). “Advances in Plant Breeding Strategies: Breeding, Biotechnology and Molecular Tools,” in Advances in Plant Breeding Strategies: Breeding, Biotechnology and Molecular Tools, 63–93. 10.1007/978-3-319-22521-0

[B20] Till B. J. Cooper J. Tai T. H. Colowit P. Greene E. A. Henikoff S. (2007). Discovery of chemically induced mutations in rice by TILLING. BMC Plant Biol. 7, 19. 10.1186/1471-2229-7-19 17428339 PMC1858691

[B21] UPOV (1989). International Union for the Protection of New Varieties of Plants. 4th meeting with International Organizations, Geneva, October 9 and 10, 1889, Revision of the Convention. Reference document: relevant extract of document IOM/IV/2. Available at https://www.upov.int/meetings/en/doc_details.jsp?meeting_id=17485&doc_id=167958.

[B22] UPOV Act (1991). International Union for the Protection of New Varieties of Plants, International Convention for the Protection of New Varieties of Plants, https://www.upov.int//upovlex/en/conventions/1991/content.html

[B23] UPOV (1992). International Union for the Protection of New Varieties of Plants, UPOV 6th Meeting with International Organizations - Essentially Derived Varieties, UPOV document 30 October 1992: 1–6, Annex 1–7.

[B24] UPOV (2017). International Union for the Protection of New Varieties of Plants, Explanatory Notes on EDV. https://www.upov.int/edocs/expndocs/en/upov_exn_edv.pdf.

[B25] UPOV (2019). International Union for the Protection of New Varieties of Plants, Explanatory Notes on EDV. https://www.upov.int/edocs/mdocs/upov/en/c_52/c_52_7_rev.pdf.

[B26] Van Der Kooij P. A. C. E. (1997). Introduction to the EC Regulation on Plant Variety Protection, KLUWER LAW International, London – The Hague – Boston.

[B27] Vosman B. Visser D. Rouppe Van Der Voort J. Smulders M. J. M. Van Eeuwijk F. (2004). The establishment of "essential derivation" among rose varieties, using AFLP. Theor. Appl. Genet. 109 (8), 1719–1725. 10.1007/s00122-004-1809-3 15490105

[B28] Zhang D. Besse C. Cao M. Q. Gandelin M. H. (2001). Evaluation of AFLPs for variety identification in modern rose. Acta Hortic. 546, 351–357.

